# The relationship between blood urea nitrogen to serum albumin ratio and cardiovascular diseases, cardiovascular mortality, and all-cause mortality in patients with diabetes mellitus

**DOI:** 10.3389/fendo.2025.1456731

**Published:** 2025-04-11

**Authors:** Hongwei Zhu, Wei Xie, Peng Wang, ShuYuan Jiang, YunQi Hua, Guo Shao, ZhiHui Li

**Affiliations:** ^1^ Department of Emergency, The Second Affiliated Hospital of Baotou Medical College, Inner Mongolia University of Science and Technology, Baotou, China; ^2^ Inner Mongolia Key laboratory of Hypoxic Translational Medicine, Baotou Medical College, Inner Mongolia, Baotou, China; ^3^ Department of Clinical Laboratory, The Second Affiliated Hospital of Baotou Medical College, Inner Mongolia University of Science and Technology, Baotou, China; ^4^ Department of Medical Oncology, Baotou Cancer Hospital, Baotou, China; ^5^ Center for Translational Medicine, the Third People’s Hospital of Longgang District, Shenzhen, China; ^6^ Medical Department, The Second Affiliated Hospital of Baotou Medical College, Inner Mongolia University of Science and Technology, Baotou, China

**Keywords:** blood urea nitrogen to serum albumin ratio, diabetes mellitus, cardiovascular disease, cardiovascular mortality, all-cause mortality

## Abstract

**Background:**

The relationship between the Blood Urea Nitrogen to Albumin Ratio (BAR) and cardiovascular diseases in diabetes, as well as cardiovascular and all-cause mortality, is not yet entirely understood. This study aimed to examine the correlation between the serum urea nitrogen to albumin ratio and cardiovascular diseases, cardiovascular mortality, and all-cause mortality in diabetes.

**Methods:**

A total of 7043 adult diabetes patients were included from the NHANES database from 2001 to 2018. The relationship between BAR and cardiovascular diseases, cardiovascular mortality and all-cause mortality in patients with diabetes mellitus was verified using baseline characteristic analysis, multivariate logistic regression analysis, multivariate Cox proportional hazards model, Kaplan-Meier (K-M) analysis, smoothed fitted curves, and subgroup analysis.

**Results:**

Results of the logistic regression analysis indicated a substantial positive association, between the BAR and the risk of cardiovascular disease in individuals with diabetes (HR, 1.09 [95% CI 1.06–1.12], p < 0.001). Cox regression analysis revealed a substantial positive association between the BAR and the risk of cardiovascular (OR, 1.13 [95% CI, 1.10–1.17], p < 0.001) and all-cause mortality (OR, 1.12 [95% CI 1.11–1.14], p < 0.001) in diabetes. The restricted cubic spline (RCS) curves indicated a non-linear relationship between BAR and the risk of cardiovascular disease, cardiovascular mortality, and all-cause mortality in diabetes (p < 0.01). The receiver operating characteristic (ROC) curves demonstrated that the BAR had superior predictive performance for cardiovascular risk (AUC: 0.648), cardiovascular mortality (AUC: 0.618), and all-cause mortality (AUC: 0.674) compared to the body mass index (BMI) (cardiovascular risk AUC: 0.525, cardiovascular mortality AUC: 0.563, all-cause mortality AUC: 0.571) and the weight-adjusted-waist index (WWI) (cardiovascular risk AUC: 0.579, cardiovascular mortality AUC: 0.497, all-cause mortality AUC: 0.570). These results underscore the enhanced ability of the BAR to discriminate between positive and negative outcomes, making it a more effective predictor than WWI. Kaplan-Meier analysis further verified the predictive capacity of BAR, for cardiovascular mortality and all-cause mortality in diabetes patients. Subgroup analysis revealed consistent associations between BAR and a variety of subgroups.

**Conclusion:**

The incidence of cardiovascular disease, cardiovascular mortality, and all-cause mortality was substantially elevated, in patients with diabetes with a higher BAR level. Cardiovascular disease, cardiovascular mortality, and all-cause mortality may be more prevalent among diabetic patients with elevated BAR levels. BAR is a novel marker for the prediction of cardiovascular disease, cardiovascular mortality, and all-cause mortality in diabetes.

## 1 Introduction

Diabetes mellitus is the most prevalent metabolic disorder, affecting more than 500 million individuals worldwide (1). Compared to those who do not have diabetes, diabetes is a significant risk factor for cardiovascular disease (CVD) (2), which is the primary cause of mortality in patients with diabetes (3). Cardiovascular diseases, such as myocardial infarction, stroke, heart failure, and coronary artery disease (2), not only significantly decrease the quality of life of patients but also place a substantial economic burden on society (4). Consequently, earlier identification of high-risk populations with cardiovascular disease and diabetes is essential for enhancing their prognosis. At present, there are no universally recognized biomarkers that can be used to predict the prognosis of diabetes in conjunction with cardiovascular disease. There is a pressing need to identify biomarkers that can early detect diabetes in conjunction with cardiovascular disease in order to facilitate early assessment, reduce mortality, and enhance outcomes.

Serum urea nitrogen (BUN) and albumin are commonly employed as clinical biochemical markers (5). Serum urea nitrogen is a critical metric for assessing renal function, as well as nutritional and fluid status. On the other hand, albumin is indicative of inflammation and nutritional status (6). A novel prognostic biomarker has been recently identified, as the ratio of serum urea nitrogen to albumin (BAR), which serves as a combined indicator of these two markers (7). Research has demonstrated that, BAR is a reliable predictor of the prognosis of a variety of diseases (7–15). Nevertheless, the predictive validity of BAR for the risk of cardiovascular disease, cardiovascular mortality, and all-cause mortality in diabetes, has not yet been verified.

The objective of this study is to examine the correlation between BAR levels and the risk of cardiovascular disease, cardiovascular mortality, and all-cause mortality in patients with diabetes. The findings have the potential to enhance the treatment and management of patients with diabetes, by offering new insights and facilitating early prediction and timely intervention.

## 2 Materials and methods

### 2.1 Data source and study population

The National Health and Nutrition Examination Survey (NHANES) is a cross-sectional survey, that is population-based and intended to gather data on the health and nutritional status of adults and children in the U.S. population (16). The protocols of NHANES have been approved by the Research Ethics Review Board of NCHS and the informed written consent were obtained from all of the participants involved in the study. Researchers worldwide are granted unrestricted access to the majority of the data contained in the NHANES database. To guarantee that the selected sample accurately represents the entire U.S. population, the survey employs a stratified, multistage probability sampling method to sample the U.S. population. Specific statistical data is accessible at https://www.cdc.gov/nchs/nhanes/index.htm. Structured interviews at home, physical examinations at the NHANES Mobile Examination Center(MEC), and laboratory tests for sample collection are administered to evaluate the nutritional and physical health status of participants.

**Figure 1:**
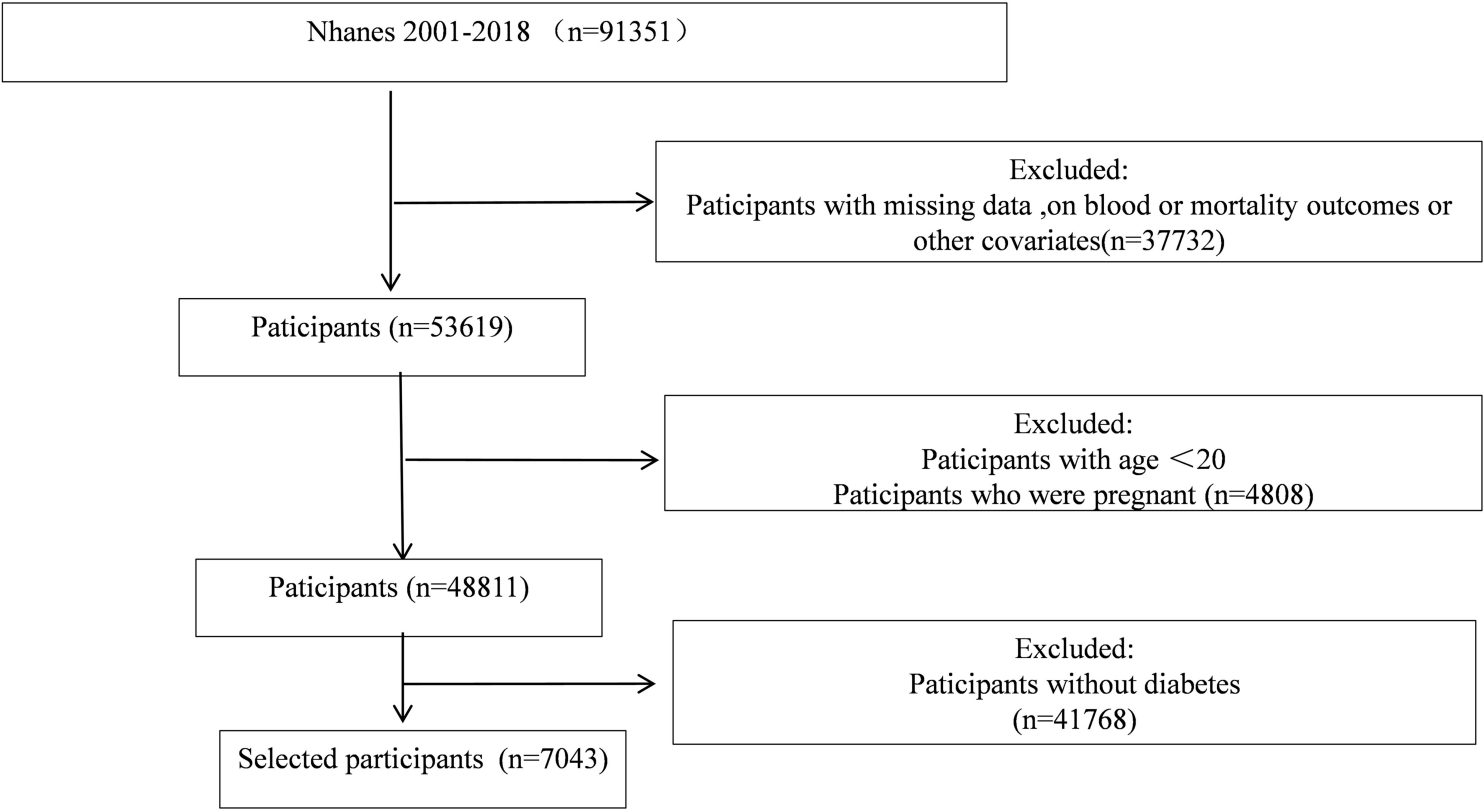
Research flow chart. Flow chart of the sample selection process.

The nine cycles of NHANES, which extend from 2001 to 2018, were employed to compile the data. The study included individuals with diabetes mellitus who were over the age of 20. The following criteria were used to diagnose diabetes mellitus: 1) self-reported physician-diagnosed diabetes mellitus; 2) currently receiving insulin injections or taking antidiabetic medication; 3) hemoglobin A1c (HbA1c) ≥6.5%; 4) fasting plasma glucose ≥7.0 mmol/L. The study included participants who satisfied any of these criteria. The following were the exclusion criteria: 1) participants who were pregnant; 2) participants who lacked data on their survival status and follow-up time; 3) participants with lacking data on their blood urea nitrogen and albumin levels; and 4) participants with invalid questionnaire data regarding their cardiovascular disease history. The final analysis comprised a total of 7043 participants. The detailed screening procedure for this study is depicted in **Figure 1**.

### 2.2 Outcome variables

The outcomes of cardiovascular diseases, such as angina, coronary heart disease, stroke, myocardial infarction, and congestive heart failure, were determined by self-reported physician diagnoses obtained through standardized medical condition questionnaires during personal interviews. Participants were asked, “Has a doctor or other health professional ever told you that you had congestive heart failure/coronary heart disease/angina/myocardial infarction/stroke?” The presence of cardiovascular disease was indicated by a positive response to any of these queries (17).

By comparing the data with the National Death Index up to December 31, 2019, mortality information was procured. The International Classification of Diseases, Tenth Revision, was employed to designate the causes of death. Cardiovascular disease-related and all-cause mortality were the two categories into which deaths were classified. Follow-up time was defined as the period from the date of NHANES interview to the date of death or to the end of follow-up.

### 2.3 Independent variables

Non-hemolyzed samples were employed to collect blood samples from fasting participants. An enzymatic conductance method was employed to measure blood urea nitrogen, while a colorimetric method and bromcresol purple were employed to measure albumin. Detailed methods for acquiring these variables can be found at www.cdc.gov/nchs/nhanes. The formula for calculating the Blood Urea Nitrogen to Albumin Ratio (BAR) was as follows: BUN (mg/dL)/serum albumin (g/dL) (18).

### 2.4 Covariates

During home interviews, standardized questionnaires were employed to collect data on age, sex, race, education level, marital status, family income, smoking status, disease status, and medication use. Race was classified as Mexican American, non-Hispanic Black, non-Hispanic White, and others. Education was classified into three levels: less than high school, high school or equivalent, and college or above. Marital status was classified into two categories: with a partner and without a partner. Smoking status was classified into three categories: never inhaled, former smoker, and current smoker. The family income-to-poverty ratio was classified as 0-1.0, 1.0-3.0, and ≥3.0. Weight, height, and blood sample collection were recorded, during physical examinations at the MEC. The body mass index (BMI) was determined by dividing weight (kg) by height (m) squared. BMI was classified as<25.0 kg/m², 25.0-30.0 kg/m², and ≥30.0 kg/m². The formula for the Weight-Adjusted Waist Index (WWI) is as follows: waist circumference (cm) divided by the square root of body weight (kg). Furthermore, the participants’ fasting blood samples were analyzed at baseline, which included total cholesterol, triglycerides, blood glucose, and glycosylated hemoglobin (HbA1c). The National Health and Nutrition Examination Survey (NHANES) Laboratory/Medical Technicians Procedure Manual was adhered to in the strictest manner, during the blood collection and analysis process. Anthropometric measurements (weight, height, and waist circumference) and blood sample collections were performed during the same visit at the MEC to ensure that the study variables were based on data collected at the same time point.

### 2.5 Statistical analysis

Continuous variables that adhered to a normal distribution are represented as mean ± standard deviation, while those that did not adhere to a normal distribution are represented as median (25th percentile, 75th percentile). The baseline differences in continuous variables were evaluated using analysis of variance (ANOVA). A one-way ANOVA test was implemented for continuous variables that were normally distributed, while the Kruskal-Wallis test was implemented for continuous variables that were not normally distributed. The Chi-square test was implemented to compare differences in categorical variables between groups.

The association between the BAR and cardiovascular diseases in diabetes was evaluated using logistic regression analysis. The hazard ratios (HRs) and 95% confidence intervals (CIs) for the risk of cardiovascular mortality and all-cause mortality in diabetic patients associated with the BAR were estimated, using Cox proportional hazards regression. The relationship between the BAR and all-cause and cardiovascular mortality in diabetic patients was analyzed using Kaplan-Meier survival analysis.

The non-linear association between the BAR and the risk of cardiovascular disease, all-cause mortality, and cardiovascular mortality in diabetic patients was analyzed using restricted cubic splines (RCS). The predictive ability of the BAR for cardiovascular disease risk, all-cause mortality, and cardiovascular mortality in diabetic patients was evaluated using receiver operating characteristic (ROC) analysis, and the area under the curve (AUC) was calculated.

The study outcomes were further analyzed in stratified analyses, to determine the impact of age, sex, marital status, and other factors. The participants were stratified by age (<65 years or ≥65 years), sex (male or female), race/ethnicity (Mexican American, non-Hispanic Black, non-Hispanic White, and other), smoking status (current or never/former), BMI (<25 kg/m², 25.0-30.0 kg/m², ≥30 kg/m²), and glycosylated hemoglobin (<7% or ≥7%). The objective of these analyses was to evaluate the reliability and robustness of the study’s results. Likelihood ratio tests were implemented to evaluate the interactions between the BAR and subgroups. In order to evaluate the stability of the results, sensitivity analyses were implemented. A preliminary step was taken to mitigate potential reverse causal bias by excluding participants who passed away within the first two years of the follow-up. Subsequently, the primary analyses were repeated using serum BAR levels as quartiles. R Software (version 4.3.2) was employed to conduct the data analysis. Statistically significant was defined as a two-tailed P-value<0.05.

## 3 Results

### 3.1 Baseline characteristics

The study encompassed a total of 7043 participants with diabetes, and the patient screening flowchart is illustrated in **Figure 1**. **Table 1** displays the baseline characteristics of participants stratified by the BAR. Of the 7043 participants with diabetes, 3400 were female, comprising 48.3% of the total, while 3643 were male, comprising 51.7%. The participants’ average age was 62.0 years. Eighty-five point seven percent of the participants were overweight, with a body mass index (BMI) ≥25 kg/m². Hypertension was present in sixty-three percent of the participants. The BAR ratio was used to divide participants into three groups based on the tertiles (T1:<2.95; T2: 2.95-4.19; T3:≥4.19). The mean BAR for all participants was 4.04± 2.30 mg/g, with the values for the different BAR tertiles groups as follows: T1: 2.32 ± 0.43 mg/g; T2: 3.52 ± 0.35 mg/g; and T3: 6.27 ± 2.73 mg/g. The reference group was T1, which was used to investigate the relationship between the BAR and the risk of cardiovascular disease, cardiovascular mortality, and all-cause mortality in diabetic patients. Age, sex, race, education level, family poverty index, marital status, BMI, waist circumference, total cholesterol, insulin use, creatinine, uric acid, blood urea nitrogen, plasma albumin, and high-density lipoprotein were all significantly different among the three groups. The prevalence of comorbid conditions, including hypertension, congestive heart failure, coronary heart disease, angina, myocardial infarction, and stroke, varied significantly between the groups. The groups did not exhibit any distinctions in glycosylated hemoglobin and blood glucose levels. Hypertension, congestive heart failure, coronary heart disease, angina, myocardial infarction, and stroke were most prevalent in the group with the highest BAR. Furthermore, this group exhibited older age, a higher proportion of females, a lower level of education, a higher proportion of individuals without long-term partners, and a higher rate of insulin use. The group with the highest BAR also exhibited a higher proportion of overweight individuals, larger waist circumferences, higher levels of total cholesterol, creatinine, uric acid, and blood urea nitrogen, and lower levels of plasma albumin and high-density lipoprotein. The mean WWI for all participants was 11.6 ± 0.76 cm/√kg, with the values for the different BAR tertiles groups as follows: T1: 11.5 ± 0.73 . $\frac{\text{cm}}{\sqrt{\text{kg}}}$ .; T2: 11.6 ± 0.76 $\frac{\text{cm}}{\sqrt{\text{kg}}}$ ; and T3: 11.7 ± 0.78 $\frac{\text{cm}}{\sqrt{\text{kg}}}$ . A total of 1589 fatalities occurred during an average follow-up of 86.7 months, with 429 of them being attributed to cardiovascular diseases. The prevalence of cardiovascular disease, as well as the risks of cardiovascular mortality and all-cause mortality, were more prevalent among individuals with higher serum BAR ratios.

**Table 1:** Baseline characteristics and outcomes by BAR categories.

	Overall(N=7043)	T1(N=2301)	T2(N=2400)	T3(N=2342)	P.value
Age (years)	62.0 (51.0,71.0)	54.0 (43.0,64.0)	62.0 (52.0,70.0)	69.0 (61.0,78.0)	<0.001
Gender:					<0.001
Female n (%)	3400 (48.3%)	1223 (53.2%)	1119 (46.6%)	1058 (45.2%)	
Male n (%)	3643 (51.7%)	1078 (46.8%)	1281 (53.4%)	1284 (54.8%)	
Race: (%)					<0.001
Mexican American	1318 (18.7%)	465 (20.2%)	475 (19.8%)	378 (16.1%)	
Other Hispanic	701 (9.95%)	265 (11.5%)	245 (10.2%)	191 (8.16%)	
Non-Hispanic white	2537 (36.0%)	644 (28.0%)	847 (35.3%)	1046 (44.7%)	
Non-Hispanic black	1731 (24.6%)	648 (28.2%)	565 (23.5%)	518 (22.1%)	
Other race	756 (10.7%)	279 (12.1%)	268 (11.2%)	209 (8.92%)	
Education: (%)					0.001
Less than high school	1293 (18.4%)	375 (16.3%)	436 (18.2%)	482 (20.6%)	
High school diploma or GED	2756 (39.1%)	935 (40.6%)	905 (37.7%)	916 (39.1%)	
More than high school	2994 (42.5%)	991 (43.1%)	1059 (44.1%)	944 (40.3%)	
Marital_status: (%)					0.001
Having a partner	5454 (77.4%)	1985 (86.3%)	2003 (83.5%)	1466 (62.6%)	
No partner	1589 (22.6%)	316 (13.7%)	397 (16.5%)	876 (37.4%)	
Income-to-poverty ratio: (%)					<0.001
<1.0	1603 (22.8%)	619 (26.9%)	480 (20.0%)	504 (21.5%)	
1.0-3.0	3293 (46.8%)	1015 (44.1%)	1140 (47.5%)	1138 (48.6%)	
≥3.0	2147 (30.5%)	667 (29.0%)	780 (32.5%)	700 (29.9%)	
Serum creatinine (mg/dl)	1.01 ± 0.70	0.79 ± 0.20	0.89 ± 0.25	1.36 ± 1.09	<0.001
Albumin(mg/dL)	4.10 (3.90,4.30)	4.20 (4.00,4.40)	4.20 (3.90,4.30)	4.00 (3.80,4.20)	<0.001
Glucose (mmol/L)	7.44 (6.00,9.60)	7.49 (6.16,9.71)	7.44 (6.00,9.49)	7.38 (5.94,9.60)	0.105
Blood urea nitrogen (mg/dl)	15.0 (11.0,19.0)	10.0 (8.00,11.0)	14.0 (13.0,16.0)	22.0 (19.0,27.0)	<0.001
Serum uric acid (mg/dl)	5.60 (4.60,6.70)	5.20 (4.30,6.20)	5.50 (4.60,6.50)	6.20 (5.20,7.40)	<0.001
TG(mg/dl)	155 (105,235)	156 (105,236)	156 (106,239)	152 (104,227)	0.136
TC(mg/dl)	188 ± 47.8	194 ± 46.9	189 ± 49.4	180 ± 46.1	<0.001
HBA1C:(%)	7.23 ± 1.76	7.26 ± 1.88	7.24 ± 1.78	7.20 ± 1.62	0.5
BAR(mg/g)	4.04 ± 2.30	2.32 ± 0.43	3.52 ± 0.35	6.27 ± 2.73	<0.001
Waist: (%)	107 (97.3,118)	106 (95.7,118)	107 (97.7,118)	108 (98.2,120)	<0.001
Weight: (%)	89.0 ± 23.6	89.2 ± 24.2	89.3 ± 22.7	88.4 ± 23.8	0.367
WWI( $\frac{\text{cm}}{\sqrt{\text{kg}}}$ )	11.6 ± 0.76	11.5 ± 0.73	11.6 ± 0.76	11.7 ± 0.78	<0.001
BMI(kg/m^2^)					0.091
<25	1006 (14.3%)	328 (14.3%)	310 (12.9%)	368 (15.7%)	
25-30	2085 (29.6%)	679 (29.5%)	733 (30.5%)	673 (28.7%)	
≥30	3952 (56.1%)	1294 (56.2%)	1357 (56.5%)	1301 (55.6%)	
Congestive heart failure: (%)					<0.001
No	6447 (91.5%)	2205 (95.8%)	2262 (94.2%)	1980 (84.5%)	
Yes	596 (8.46%)	96 (4.17%)	138 (5.75%)	362 (15.5%)	
Coronary heart disease: (%)					<0.001
No	6369 (90.4%)	2183 (94.9%)	2215 (92.3%)	1971 (84.2%)	
Yes	674 (9.57%)	118 (5.13%)	185 (7.71%)	371 (15.8%)	
Angina:(%)					<0.001
No	6584 (93.5%)	2190 (95.2%)	2263 (94.3%)	2131 (91.0%)	
Yes	459 (6.52%)	111 (4.82%)	137 (5.71%)	211 (9.01%)	
Heart attack:(%)					<0.001
No	6348 (90.1%)	2148 (93.4%)	2217 (92.4%)	1983 (84.7%)	
Yes	695 (9.87%)	153 (6.65%)	183 (7.62%)	359 (15.3%)	
Stroke: (%)					<0.001
No	6465 (91.8%)	2193 (95.3%)	2223 (92.6%)	2049 (87.5%)	
Yes	578 (8.21%)	108 (4.69%)	177 (7.38%)	293 (12.5%)	
Insulin ues:(%)					<0.001
No	5659 (80.3%)	2017 (87.7%)	2011 (83.8%)	1631 (69.6%)	
Yes	1384 (19.7%)	284 (12.3%)	389 (16.2%)	711 (30.4%)	
Smoking status:(%)					0.026
No	3552 (50.4%)	1132 (49.2%)	1264 (52.7%)	1156 (49.4%)	
Yes	3491 (49.6%)	1169 (50.8%)	1136 (47.3%)	1186 (50.6%)	
Hypertension: (%)					<0.001
No	2605 (37.0%)	1102 (47.9%)	904 (37.7%)	599 (25.6%)	
Yes	4438 (63.0%)	1199 (52.1%)	1496 (62.3%)	1743 (74.4%)	
Time(month)	86.7 ± 52.6	97.5 ± 51.0	88.8 ± 53.1	73.9 ± 50.9	<0.001
Cardiovascular diseases:(%)					<0.001
No	5351 (76.0%)	1953 (84.9%)	1912 (79.7%)	1486 (63.5%)	
Yes	1692 (24.0%)	348 (15.1%)	488 (20.3%)	856 (36.5%)	
Cardiovascular Mortality:(%)					<0.001
No	6614 (93.9%)	2243 (97.5%)	2284 (95.2%)	2087 (89.1%)	
Yes	429 (6.09%)	58 (2.52%)	116 (4.83%)	255 (10.9%)	
All-cause Mortality:(%)					<0.001
No	5454 (77.4%)	1985 (86.3%)	2003 (83.5%)	1466 (62.6%)	
Yes	1589 (22.6%)	316 (13.7%)	397 (16.5%)	876 (37.4%)	

### 3.2 Relationship between the BAR ratio and the risk of cardiovascular disease in diabetes

Cardiovascular disease was present in 1692 of the 7043 participants. In order to evaluate the association between the BAR and the risk of cardiovascular disease in diabetes, we developed three logistic regression models. In the unadjusted model, the BAR was significantly associated with cardiovascular risk when it was considered as a continuous variable (OR: 1.25; 95% CI: 1.22-1.28; P<0.001). In a multivariate logistic regression model that was adjusted for potential confounders, such as age, sex, race, marital status, family poverty index, education, insulin use, hypertension, and waist circumference, the BAR remained significantly associated with the risk of overall cardiovascular disease in diabetes (OR: 1.09; 95% CI: 1.06-1.12; P<0.001).

In the unadjusted model, the T3 BAR group had a significantly higher risk of cardiovascular disease than the T1 group, when the BAR was considered as a categorical variable (OR: 3.23; 95% CI: 2.81-3.73; P<0.001). The difference remained statistically significant after full adjustment (T3: OR: 1.35; 95% CI: 1.15-1.59; P<0.01). The risk of cardiovascular disease in diabetes was substantially correlated with the BAR, which implies that it could be employed as a predictive indicator for cardiovascular disease. The logistic regression analysis results are reported in **Table 2**.

**Table 2:** Logistic regression analysis of the association between BAR ratio and the risk of cardiovascular disease in diabetes.

Variable	Model 1			Model 2			Model 3		
	OR	OR 95% CI	P-value	OR	OR 95% CI	P-value	OR	OR 95% CI	P-value
BAR	1.25	1.22-1.28	<0.001	1.14	1.12-1.18	<0.001	1.09	1.06-1.12	<0.001
BAR									
T1	Reference			Reference			Reference		
T2	1.43	1.23-1.67	<0.001	1.04	0.89-1.22	0.615	0.94	0.80-1.11	0.482
T3	3.23	2.81-3.73	<0.001	1.74	1.49-2.04	<0.001	1.35	1.15-1.59	<0.001

Model 1: no covariates were adjusted. Model 2: age, gender, and race were adjusted. Model 3: age, gender, race, marital_status, income-to-poverty ratio, heart attack:congestive heart failure insulin use, and total cholesterol were adjusted.

### 3.3 Relationship between the BAR ratio and cardiovascular and all-cause mortality in diabetes

The Cox proportional hazards regression model revealed that the BAR ratio, when considered as a continuous variable, was robustly linked to cardiovascular mortality (HR: 1.21; 95% CI: 1.19-1.23; P<0.001) and all-cause mortality (HR: 1.19; 95% CI: 1.18-1.20; P<0.001) in the unadjusted analysis. Upon multivariate adjustment for confounders including age, gender, race, marital status, family poverty index, education level, and insulin use, the BAR ratio continued to demonstrate significant associations with cardiovascular mortality (HR: 1.13; 95% CI: 1.10-1.17; P<0.001) and all-cause mortality (HR: 1.12; 95% CI: 1.11-1.14; P<0.001) in the diabetic cohort. Moreover, when the BAR ratio was stratified into tertiles (T1, T2, T3), the highest tertile (T3) exhibited a significantly elevated risk of cardiovascular mortality (HR: 5.98; 95% CI: 4.50-7.96; P<0.001) and all-cause mortality (HR: 3.80; 95% CI: 3.34-4.32; P<0.001) compared to the lowest tertile (T1) in the unadjusted model. Notably, even after comprehensive adjustment, the differences between the highest and lowest tertiles remained statistically significant for both cardiovascular mortality (T3: HR: 2.14; 95% CI: 1.58-2.90; P<0.001) and all-cause mortality (T3: HR: 1.50; 95% CI: 1.31-1.73; P<0.001) in diabetic patients. These findings suggest that the BAR ratio is positively correlated with cardiovascular and all-cause mortality in diabetes, underscoring its potential as a prognostic marker for these critical outcomes. The results of the Cox proportional hazards regression analysis are detailed in **Table 3**.

**Table 3:** Cox regression analysis of the association between BAR ratio and cardiovascular mortality, all-cause mortality in diabetes.

Variable	Model 1			Model 2			Model 3		
	HR	HR 95% CI	P-value	HR	HR 95% CI	P-value	HR	HR 95% CI	P-value
Cardiovascular Mortality									
BAR	1.21	1.19-1.23	<0.001	1.17	1.14-1.20	<0.001	1.13	1.10-1.17	<0.001
BAR									
T1	Reference			Reference			Reference		
T2	2.13	1.55-2.92	<0.001	1.27	0.93-1.75	0.138	1.32	0.954-1.813	0.094
T3	5.98	4.50-7.96	<0.001	2.39	1.77-3.23	<0.001	2.14	1.580-2.900	<0.001
All-cause Mortality									
BAR	1.19	1.18-1.20	<0.001	1.15	1.13-1.16	<0.001	1.12	1.11-1.14	<0.001
BAR									
T1	Reference	Reference	Reference						
T2	1.34	1.16-1.56	<0.001	0.84	0.72-0.98	<0.001	0.86	0.74-0.99	<0.05
T3	3.80	3.34-4.32	<0.001	1.64	1.43-1.89	<0.001	1.50	1.31-1.73	<0.001

Model 1: no covariates were adjusted.

Model 2: age, gender, and race were adjusted.

Model 3: age, gender, race, marital_status, income-to-poverty ratio, heart attack:congestive heart failure insulin use, and total cholesterol were adjusted.

### 3.4 The results of the restricted cubic spline analysis

**Figure 2:**
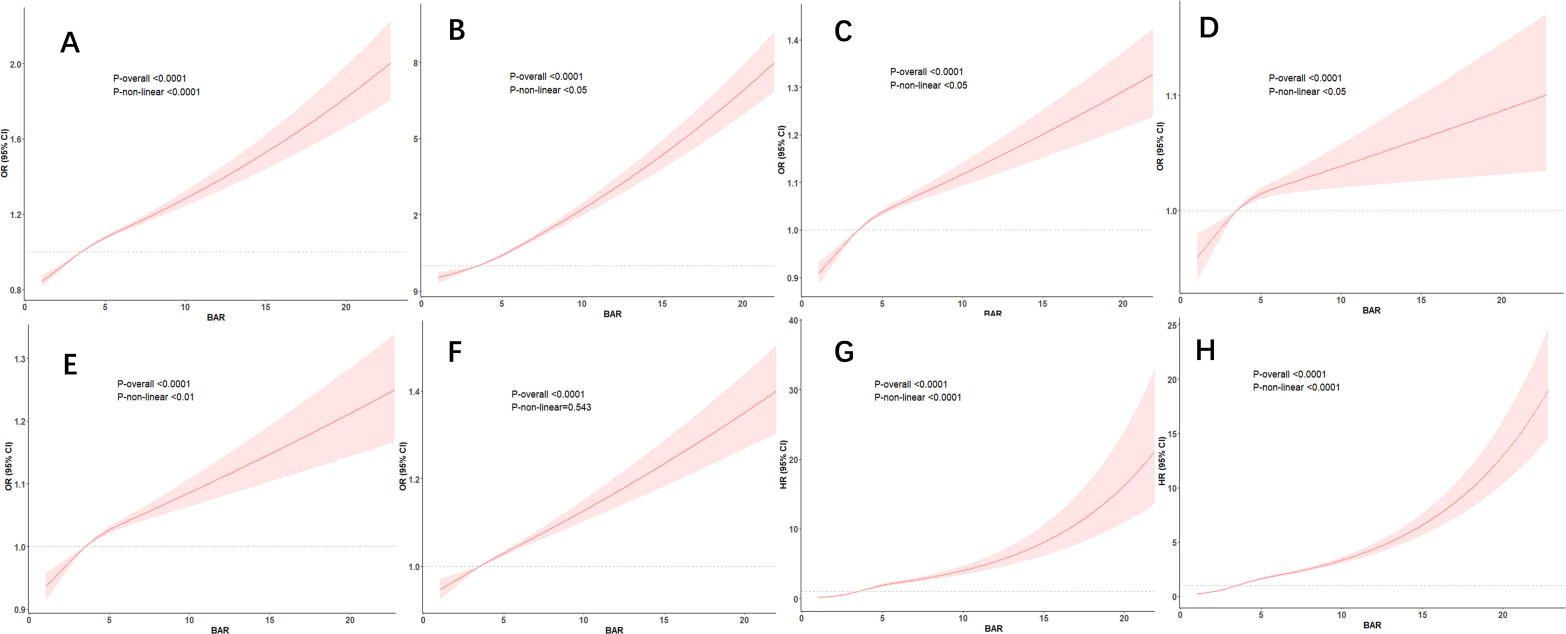
RCS curves for BAR and the risk of cardiovascular disease, cardiovascular mortality, and all-cause mortality in diabetes. —associations between BAR with cardiovascular diseases **(A)**, congestive heart failure **(B)**, coronary heart disease **(C)**, Angina **(D)**, Heart attack **(E)**, Stroke **(F)**, cardiovascular mortality **(G)** and all-cause mortality **(H)** among participants with diabetes in NHANES 2001–2018.

The results of the Restricted Cubic Spline (RCS) Analysis indicate a nonlinear relationship, between the BAR Ratio and the risk of cardiovascular disease, cardiovascular mortality, and all-cause mortality in diabetes (p<0.001). The cumulative risk of cardiovascular disease, cardiovascular mortality, and all-cause mortality in diabetes increased (p<0.001), in conjunction with the increase in the BAR ratio (**Figure 2**).

### 3.5 ROC curve analysis

ROC curve analysis was implemented, to evaluate the predictive value of the BAR for the risk of cardiovascular disease, cardiovascular mortality, and all-cause mortality in diabetes.

**Figure 3:**
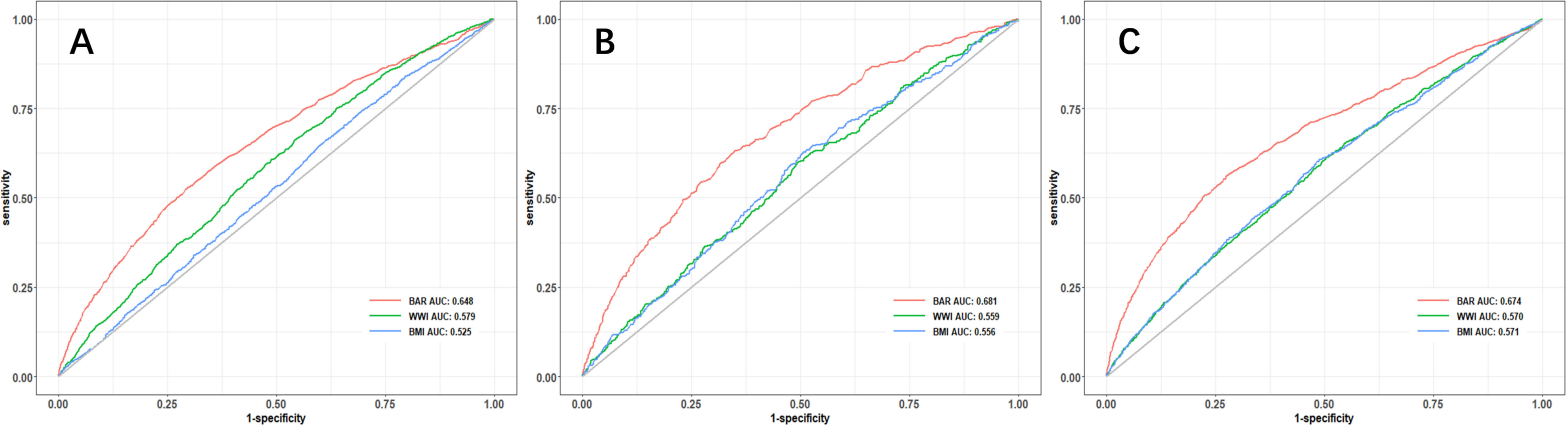
ROC curve analysis of the predictive value of BAR ratio for the risk of cardiovascular disease **(A)**, cardiovascular mortality **(B)**, and all-cause mortality **(C)** in diabetes.

According to the ROC analysis, the BAR’s area under the curve (AUC) for the prediction of cardiovascular disease, cardiovascular mortality, and all-cause mortality in diabetes was 0.648 (95% CI, 0.633-0.664), 0.618 (95% CI, 0.590-0.645), and 0.674 (95% CI, 0.658-0.690), respectively. (**Table 4**) The BAR has a superior predictive value for cardiovascular risk, cardiovascular mortality, and all-cause mortality in diabetes when compared to BMI and WWI, respectively. The ROC curves are illustrated in **Figure 3**, and the specific parameters of the ROC curve.

**Table 4:** ROC curve analysis of BAR for predicting cardiovascular disease, all-cause mortality, and cardiovascular mortality in diabetes.

	AUC	95%CI	Threshold	Specificity	Sensitivity	Youden
Cardiovascular diseases						
BARROC	0.648	0.633-0.664	4.1	0.703	0.53	0.23
BMIROC	0.525	0.509-0.54	28.9	0.386	0.663	0.05
WWIROC	0.579	0.564-0.579	11.5	0.457	0.662	0.12
Cardiovascular Mortality						
BARROC	0.618	0.59-0.645	0.538	0.783	0.402	0.19
BMIROC	0.563	0.536-0.59	29.1	0.622	0.482	0.1
WWIROC	0.497	0.471-0.524	9.47	0.64	0.39	0.03
All-cause Mortality						
BARROC	0.674	0.658-0.69	4.14	0.719	0.666	0.29
BMIROC	0.571	0.555-0.587	31.1	0.518	0.601	0.12
WWIROC	0.57	0.554-0.586	11.5	0.495	0.614	0.11

### 3.6 Survival analysis

**Figure 4:**
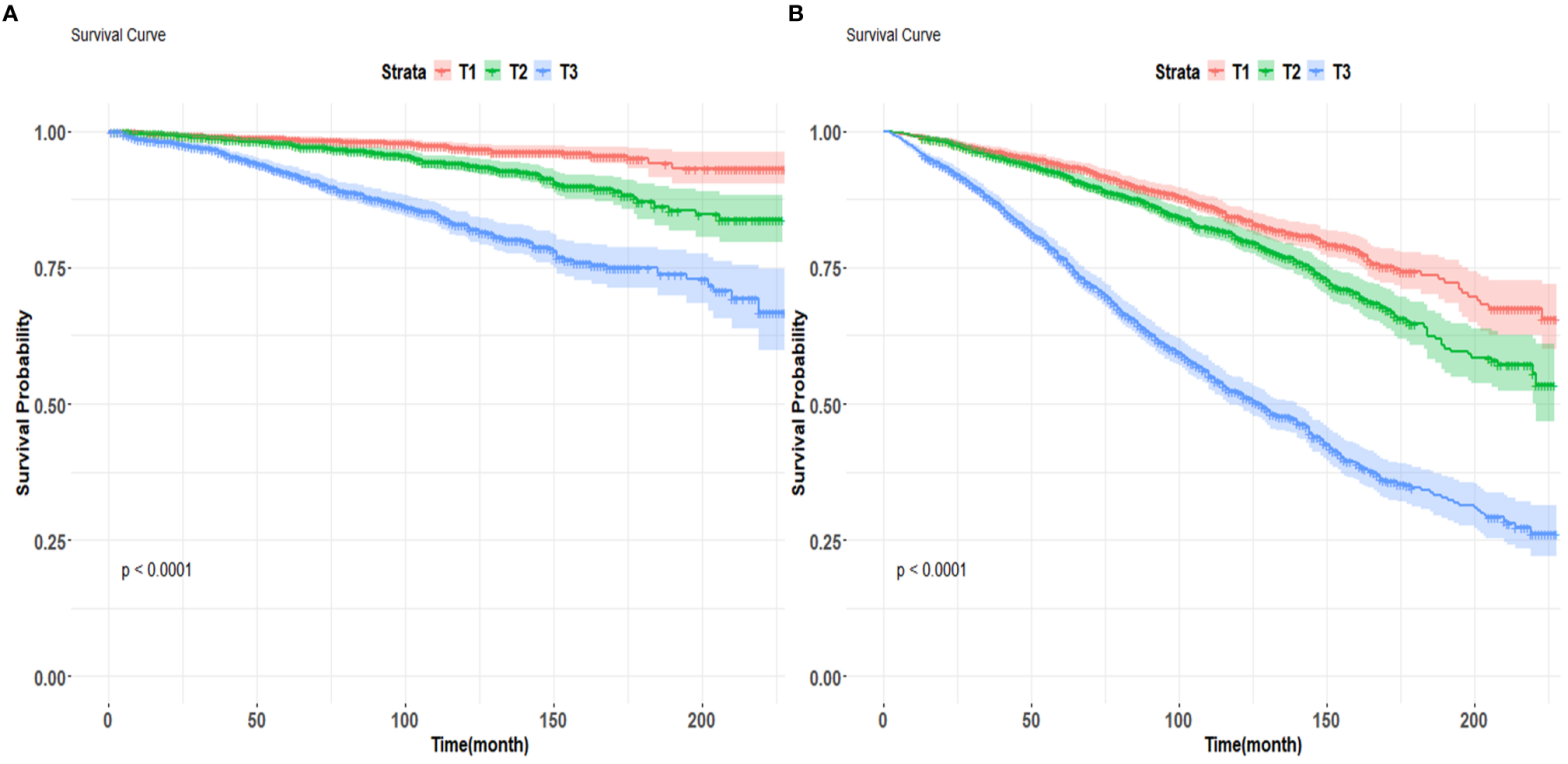
Kaplan-Meier survival curves for cardiovascular mortality **(A)** and all-cause mortality **(B)** by BAR tertile groups figure.

Kaplan-Meier survival curves were employed to investigate the correlation between cardiovascular mortality and all-cause mortality with BAR tertiles. The Kaplan-Meier analysis demonstrated substantial disparities in all-cause mortality and cardiovascular mortality among the BAR tertile groups. In comparison to the T1 group, the mortality rate in the T3 group was substantially elevated (P<0.001) (**Figure 4**).

### 3.7 Subgroup analysis

Subgroup and interaction analyses were conducted based on gender, marital status, race, education level, poverty index, age group (<65 years, ≥65 years), smoking status, BMI, and HbA1c levels (<7%, ≥7%). The poverty index and BMI were found to interact with the BAR and diabetes cardiovascular risk in the subgroup analysis (interaction P<0.05).

A stronger positive association between BAR and cardiovascular disease was observed in participants with middle poverty index (OR 1.27, 95% CI: 1.23-1.32) or high poverty index (OR 1.28, 95% CI: 1.22-1.35) and BMI of 25-30 (OR 1.35, 95% CI: 1.28-1.43) (P< 0.05).

The interaction with race and age group was observed in the context of BAR and diabetes cardiovascular mortality. Compared to other races, other Hispanic diabetic patients (HR 1.36, 95% CI: 1.19-1.56) had a higher risk of cardiovascular mortality. Diabetic patients under 65 years of age (HR 1.22, 95% CI: 1.16-1.29) had a higher risk of cardiovascular mortality compared to those aged 65 and above (HR 1.12, 95% CI: 1.08-1.16).

The interaction with race and HbA1c level was observed in the subgroup analysis of BAR and diabetes all-cause mortality. Patients of other races (HR 1.43, 95% CI: 1.27-1.62) had a higher risk of all-cause mortality. Diabetic patients with HbA1c levels less than 7% (HR 1.38, 95% CI: 1.33-1.43) had a higher risk of all-cause mortality.

**Figure 5:**
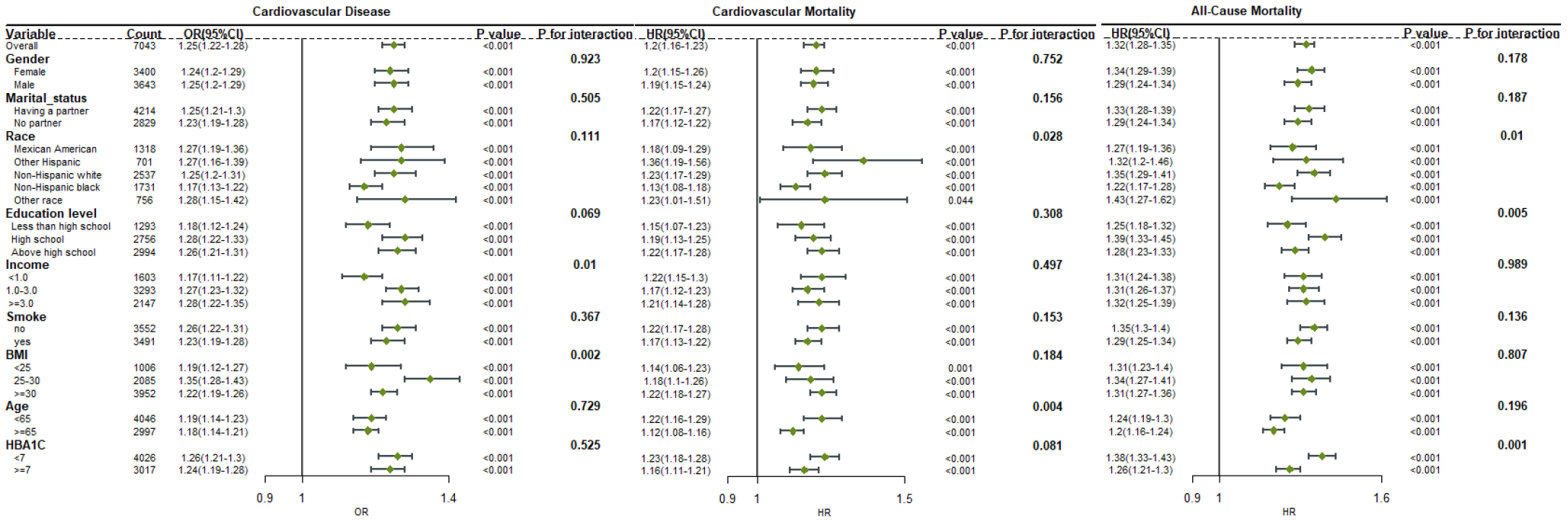
Forest plot for the association between BAR ratio and the risk of cardiovascular disease, cardiovascular mortality, and all-cause mortality in diabetes.

The stratified results were in accordance with the overall results. In all subgroups, BAR was consistently correlated with diabetes cardiovascular disease, cardiovascular mortality, and all-cause mortality, demonstrating a significant association (**Figure 5**).

## 4 Discussion

In this study, we examined the correlation between BAR levels and cardiovascular disease, cardiovascular mortality, and all-cause mortality in diabetic patients. This study is the first to show that elevated BAR levels are a robust independent predictor of cardiovascular disease, cardiovascular mortality, and all-cause mortality in patients with diabetes. The independent association between the BAR and cardiovascular disease, cardiovascular mortality, and all-cause mortality in diabetic patients persisted, even after adjusting for potential confounding risk factors. Consequently, the BAR necessitates attention in clinical practice and can function as an independent predictor of cardiovascular disease, cardiovascular mortality, and all-cause mortality in diabetic patients. This study introduces a novel, simple, and efficient biomarker for cardiovascular disease, cardiovascular mortality, and all-cause mortality in diabetic patients.

Blood urea nitrogen is a protein metabolite that is produced by the liver and excreted by the kidneys. It is frequently employed as a biomarker to evaluate renal function in conjunction with other measurements, such as creatinine. The development of diabetes is positively correlated with BUN levels, as demonstrated by existing research (19, 20). A nonlinear association between BUN and all-cause mortality and cardiovascular mortality in patients with diabetes is suggested by a large cohort study of the U.S. population. By ensuring that patients with diabetes maintain appropriate BUN levels, it may be possible to prevent all-cause, or cardiovascular mortality (21–23). Additionally, clinical research has demonstrated that BUN levels are elevated in patients with cardiovascular disease and are positively correlated with all-cause and cardiovascular mortality (21). Consequently, blood urea nitrogen is regarded as an effective predictor of cardiovascular prognosis (24).

Serum albumin is a multifunctional circulating protein that is involved in the regulation of numerous critical physiological functions, including the maintenance of colloid osmotic pressure and microvascular integrity, the regulation of metabolism and vascular function, the provision of binding ligands for substances, antioxidant activity, and anticoagulant impacts (25). Furthermore, inflammatory processes are negatively correlated, with serum albumin’s ability to modulate neutrophil adhesion and partial cell signaling (26, 27). In a variety of clinical settings, serum albumin is regarded as a dependable biomarker for risk prediction (28). It has been verified that an elevated risk of cardiovascular mortality and all-cause mortality is linked to reduced serum albumin levels (29, 30). In elderly patients, hypoalbuminemia is linked to an elevated risk of cardiovascular disease (6). Patients who exhibit decreased serum albumin levels may be susceptible to cardiovascular disease, even when their levels are within the normal range (6). A prospective community cohort study has demonstrated that cerebrovascular mortality is independently predicted by reduced albumin levels (31). Serum albumin’s prognostic value for cerebrovascular and cardiovascular diseases is restricted by its sensitivity to a variety of factors, including liver function, catabolism, and extravascular leakage (32).

Blood urea nitrogen and serum albumin are clinically significant, and the Blood Urea Nitrogen to Albumin Ratio consolidates this significance by taking into account the nutritional status, protein metabolism, and liver and kidney function. In comparison to BUN and albumin in isolation, the BAR is theoretically capable of providing a more accurate prediction of the ultimate prognosis of cardiovascular disease. BUN and albumin are both serological markers that offer the advantages of swift detection and cost-effectiveness. The BAR has the potential to address the limitations of relying exclusively on albumin or BUN for the prediction of cardiovascular-related diseases. The prognostic relevance of BAR in a variety of conditions, including sepsis, cardiac surgery, pneumonia, chronic heart failure, acute kidney injury, and a host of other diseases, is currently supported by evidence (7–10, 12, 15, 33–36). An elevated BAR has been linked to an increased risk of short-term mortality (14, 36). Nevertheless, the function of BAR in the long-term prognosis of diabetic patients has not yet been elucidated by existing prospective studies. There is a scarcity of published research on the relationship between BAR and the risk of cardiovascular disease, cardiovascular mortality, and all-cause mortality in diabetic patients.

In our study, we enrolled 7043 patients with diabetes and found that BAR is associated with the risk of cardiovascular disease, cardiovascular mortality, and all-cause mortality in diabetic patients, regardless of whether it is treated as a continuous or categorical variable. Remarkably, we discovered that BAR has a more robust predictive ability for cardiovascular disease, cardiovascular mortality, and all-cause mortality in diabetic patients than both WWI and BMI. Clinicians can predict mortality risk by conducting an early BAR assessment, and patients with elevated BAR levels should be cautious of unfavorable prognostic outcomes. In order to improve the prognosis of diabetic patients, clinicians can implement more proactive management and intervention strategies at an early stage.

The underlying pathophysiological mechanism of the association between BAR and adverse outcomes in diabetic patients remains unclear. It has been speculated that impaired renal function, inflammation, malnutrition, and endothelial dysfunction may be involved (37). Firstly, BUN plays a key role in the assessment of renal function (37). Elevated BUN levels reflect not only increased protein catabolism and urea cycle disorders, but also hemodynamic and neurohormonal changes (38). These are recognized factors influencing cardiovascular risk and adverse outcomes in patients with diabetes. Secondly, increasing evidence shows that inflammation is an important factor in the progression of diabetes and related complications (39). Elevated BUN levels can lead to inflammation and vascular damage. In the physiological state, serum albumin exerts anti-inflammatory effects through various mechanisms, including binding of inflammatory mediators, regulation of the inflammatory response, and antioxidation (40). These protective effects were attenuated when serum albumin levels were reduced. Thirdly, elevated BAR may also indicate poor nutritional status (7), which is an important risk factor for poor outcomes in patients with diabetes. Reduced serum albumin levels resulting in elevated BAR indicate poor nutritional status, a risk factor for cardiovascular disease. Lastly, increased BUN can lead to endothelial dysfunction, while decreased serum albumin can lead to increased blood viscosity and impaired endothelial function (41). Endothelial dysfunction is a contributing factor to adverse outcomes in diabetic patients (42).

Limitations of this study include: Firstly, this study is retrospective in nature. Despite the utilization of multivariate adjustment and subgroup analysis by researchers, residual confounding remains a possibility. Secondly, the type of diabetes cannot be directly extracted from this database. A deeper investigation is not feasible. Thirdly, only the results of serum urea nitrogen and albumin measurements that were taken once in patients were utilized. This may restrict its ability to accurately represent long-term conditions and necessitate the examination of additional time points and dynamic changes. Lastly, our study was conducted on the U.S. populace; consequently, the generalizability of our findings to other populations remains uncertain. Therefore, further prospective randomized controlled trials are required to verify the results of this study.

## 5 Conclusion

As the BAR increases, the risk of cardiovascular disease, cardiovascular mortality, and all-cause mortality in patients with diabetes also increases. The BAR level exhibits a significant predictive capacity for cardiovascular disease risk, cardiovascular mortality, and all-cause mortality in diabetic patients.

## Data Availability

The datasets presented in this study can be found in online repositories. The names of the repository/repositories and accession number(s) can be found below: https://www.cdc.gov/nchs/nhanes/index.htm.
